# Nanogels for Pharmaceutical and Biomedical Applications and Their Fabrication Using 3D Printing Technologies

**DOI:** 10.3390/ma11020302

**Published:** 2018-02-16

**Authors:** Hyunah Cho, Udayabhanu Jammalamadaka, Karthik Tappa

**Affiliations:** 1 Pharmaceutical Sciences, School of Pharmacy and Health Sciences, Fairleigh Dickinson University, 230 Park Ave, Florham Park, NJ 07932, USA; 2 Mallinckrodt Institute of Radiology, Washington University School of Medicine, 216 S Kingshighway Blvd, St. Louis, MO 63110, USA; ujammalamadaka@wustl.edu (U.J.); kktappa@wustl.edu (K.T.)

**Keywords:** hydrogels, nanogels, 3D printing

## Abstract

Nanogels are hydrogels formed by connecting nanoscopic micelles dispersed in an aqueous medium, which give an opportunity for incorporating hydrophilic payloads to the exterior of the micellar networks and hydrophobic payloads in the core of the micelles. Biomedical and pharmaceutical applications of nanogels have been explored for tissue regeneration, wound healing, surgical device, implantation, and peroral, rectal, vaginal, ocular, and transdermal drug delivery. Although it is still in the early stages of development, due to the increasing demands of precise nanogel production to be utilized for personalized medicine, biomedical applications, and specialized drug delivery, 3D printing has been explored in the past few years and is believed to be one of the most precise, efficient, inexpensive, customizable, and convenient manufacturing techniques for nanogel production.

## 1. Introduction

Hydrogels, three-dimensional polymeric networks, are known to be formed in response to environmental stimuli, such as pH, temperature, hydrophilicity of the medium, and the presence of ions or enzymes [1,2,3,4]. Polymers containing hydrophilic domains dispersed uniformly in the medium are called “sols”. Continuation of physical or chemical linking processes of the dispersed polymers results in increasing the size of polymer networks, decreasing solubility of polymers, and increasing viscosity of the medium, which refers to “gelation (a sol-to-gel transition)” [5]. Here, gels can be constructed either via physical (often reversible) or chemical (often permanent) associations of polymers. Physically-assembled gels are built with polymer networks tied via hydrogen bonds, ionic interactions, hydrophobic associations, or agglomerations. For example, agarose is a copolymer altering *O*-3-linked β-_D_-galactopyranosyl and *O*-4-linked 3,6-anhydro-α-_L_-galactopyranosyl residues [6]. Hydrogen bond formation establishes intermolecular interactions between the ring *O*-3,6-atom and OH-2 which oriented at axial configuration of 3,6-anhydro-α-_L_-galactopyranosyl residue of the adjacent molecules which, in turn, leads to a formation of thermoreversible gels. Chemically-established gels are commonly prepared via a three-dimensional polymerization using a water-soluble monomeric polymer and a multi-functional cross-linker, or via radiation, activating a crosslinking process of the ready-made, branched hydrophilic polymers [2]. For example, Cho et al. formulated an in situ crosslinkable hydrogel based on adipic dihydrazide-modified hyaluronic acid (HA) and oxidized HA which instantly crosslink via the hydrazone bonds as they are mixed together [7].

Hydrogels can swell by absorbing large amounts of water or biological fluids while maintaining their network structure. Hydrogels are biocompatible (often biodegradable) soft materials closely resembling the consistency and textures of soft tissues. The unique properties of hydrogels and their resemblance to living tissues opened opportunities for biomedical applications [2]. For example, hydrogels have been used as one of the major ingredients for contact lenses, wound dressings, and tissue engineering scaffolds. In recent decades, hydrogels have earned their reputation in the pharmaceutical field, aiming for efficient peroral, rectal, vaginal, ocular, and transdermal drug delivery. Lately, there has been an increasing interest in combining nanoparticles and hydrogels together, creating “nano-in-hydrogels (known as nanogels)” to obtain maximum utility. This term “nanogels” defines hydrogels formed by physically or chemically crosslinking polymer networks confined to a nanoscopic size [8]. In this review, we summarized the recent advances and challenges in nanogel development for biomedical and pharmaceutical applications, and reviewed 3D printing technologies as promising tools for preparing not only some of the conventional pharmaceutical dosage forms, but also nanogels for biomedical and pharmaceutical applications.

## 2. Nanogels for Delivery of Poorly Water Soluble Drugs

**Figure 1:**
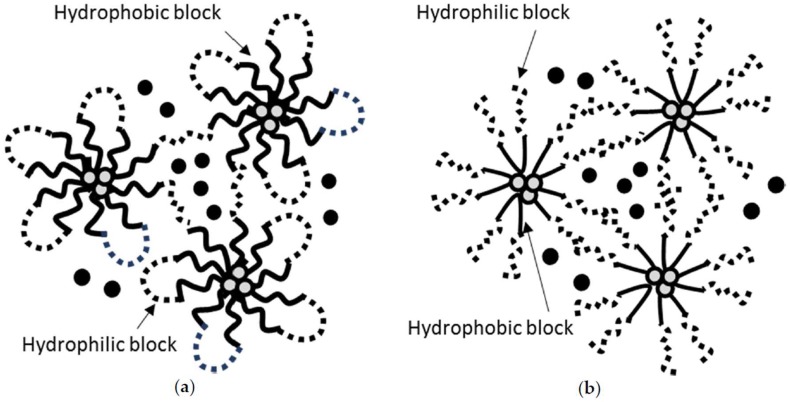
Schematic illustration of nanogels loaded with hydrophilic (solid circle) and hydrophobic (light grey circle) drugs using (**a**) BAB (hydrophobic-hydrophilic-hydrophobic) block copolymers or (**b**) ABA (hydrophilic-hydrophobic-hydrophilic) block copolymers.

Nanogels are hydrogels formed by connecting nanoscopic micellar networks. Due to this unique structure consisting of micelles (hydrophobic core-hydrophilic exterior), nanogels are capable of incorporating hydrophilic compounds into the exterior of the micellar networks and hydrophobic compounds into the core of the micelles (Figure 1) [3]. Poly (D,L-lactide-co-glycolide)-*block*-poly(ethylene glycol)-*block*-poly(D,L-lactide-co-glycolide)(PLGA-*b*-PEG-*b*-PLGA; BAB block copolymer) nanogels (Figure 1a) have been widely used to locally deliver hydrophobic drugs at a slow elution rate [9,10]. Cho et al. successfully prepared a thermosensitive PLGA-*b*-PEG-*b*-PLGA nanogels which entrain three poorly water-soluble compounds, paclitaxel (a mitotic inhibitor), rapamycin (an mTOR inhibitor), and LS301 (cysteine-glycine-arginine-aspartic acid-serine-proline-cysteine-lysine-cypate, a cypate-based angiogenesis-targeting fluorescence imaging agent). The z-average diameters of PLGA-*b*-PEG-*b*-PLGA nanogels carrying three payloads were ca. 140 nm at 10 °C, ca. 140 nm and 450 nm (bimodal) at 25 °C, and ca. 800 nm at 37 °C. This result proved the presence of micelles at 10 °C and the association of micellar networks leading to a gelation at 37 °C. Three payloads were released from the nanogel matrix at an extended elution rate with the release half-lives of ca. 101 h, 102 h, and 97 h for paclitaxel, rapamycin, and LS301, respectively. PEG-*b*-poly(D,L-lactide) (PEG-*b*-PLA) micelles (a liquid formulation) loaded with three payloads showed much shorter release half-lives of ca. 18 h, 20 h, and 20 h for paclitaxel, rapamycin, and LS301, respectively. This report proved that a nanogel is capable of loading hydrophobic drugs in despite of the rich water content in the hydrogel matrix and considered to be a great local drug delivery system eluting hydrophobic payloads at the slower rate in comparison to a liquid micellar formulation. It has been reported that PLGA-*b*-PEG-*b*-PLGA nanogels termed Regel^®^ were able to load various compounds including insulin, porcine growth hormone, recombinant hepatitis B surface antigen, paclitaxel, and cyclosporine A [11].

One of the examples of nanogels constructed with BAB block copolymer is poly (lactide)-*b*-PEG-*b*-poly (lactide) (PLA-*b*-PEG-*b*-PLA). Asadi et al. prepared and characterized PLA-*b*-PEG-*b*-PLA nanogels for controlled release of naltrexone, a hydrophobic narcotic antagonist [12]. The size of nanogels was controlled between 129 nm and 200 nm by modifying concentrations/repeating units of polymer blocks. PLA-*b*-PEG-*b*-PLA nanogels carrying naltrexone exhibited excellent long term stability without noticeable aggregations. Most importantly, PLA-*b*-PEG-*b*-PLA nanogels were able to slowly release naltrexone for 35 days. Poloxamer (also known as Pluronics) is one of the ABA triblock copolymers which carry poly (propylene oxide) (PPO) center block and poly (ethylene oxide) (PEO) side chains. Poloxamer is capable of forming micellar networks at an elevated temperature via hydrophobic interactions among the PPO blocks (Figure 1b). In particular, Pluronic F-127 (PF-127) has been widely studied in the design of dermal/transdermal, buccal, rectal, and injectable drug delivery systems, with an attempt of easy application and extended release of payloads [13]. Amiji et al. developed in situ PF-127 nanogels (20% *w*/*w*) slowly eluting paclitaxel to deliver paclitaxel intratumorally at 20 mg/kg in B16F1 melanoma-bearing mice [14]. Nie et al. also develop in situ PF-127 nanogels (18% *w*/*w*) carrying paclitaxel-loaded liposomes for controlled release of paclitaxel and improved anticancer efficacy [15]. Using a dialysis method in vitro, Taxol^®^ (paclitaxel dissolved in cremophor/ethanol (1:1, *v*/*v*) eluted almost 100% paclitaxel in 12 h. Liquid liposomal paclitaxel retarded drug release by releasing 50% payloads in 12 h. Reduction in drug release was observed from PF-127 nanogels carrying free paclitaxel (ca. 30% for 12 h) due to the increased viscosity of the gel matrix. More significant reduction in drug release was observed from PF-127 nanogels carrying paclitaxel-loaded liposomes (ca. 20% for 12 h) due to the liposomes serving as a reservoir in addition to the gel matrix being viscous.

## 3. Smart Nanogels for Pharmaceutical and Biomedical Applications

The size of the drug delivery carriers has been recognized to be a leading factor for passive targeting of cancer relying on the enhanced permeation and retention (EPR) effect [16]. Briefly, thanks to the unique physiological properties of cancer (hypervascularization, increased vascular permeability, enlarged endothelial cell junctions, stimulated extravasation, and lack of lymphatic drainage), drug delivery systems at 20–100 nm in size, such as micelles and liposomes, have been known to circulate longer in the plasma and effectively penetrate cancer cell membranes. Great efforts have been made to achieve higher drug concentrations in the plasma using nano-sized drug delivery systems loaded with anticancer drugs for superior anticancer efficacy and minimal off-targeting-associated side effect toxicity. Similarly, nanogels, nanosized hydrogels, have been tailored to fully utilize the EPR effect. Blackburn et al. successfully fabricated monodispersed core/shell nanogels at the wide particle size range of 40–140 nm using three components: *N*-isopropylacrylamide or *N*-isopropylmethacrylamide as the main monomer, acrylic acid or 4-acrylamidofluorescein as the co-monomer, and acrylamide as the crosslinker [17]. Ayame et al. constructed cationic nanogels at an average particle size of ca. 50 nm using cholesteryl group-bearing pullulan [18]. Cationic nanogels appeared to show higher internalization efficacy of the loaded protein presumably due to the formation of colloidally-stable nanoparticles carrying the protein. They also found that there was an involvement of macropinocytosis for the nanogel-protein uptake.

Nanogels are capable of active delivery of the payloads by utilizing active targeting moieties. Murphy et al. designed phospholipid-coated nanogels conjugated with the cyclic peptides, such as cRGDfK [19]. Encapsulation of docetaxel formed nanogels at the average diameter of ca. 90 nm. RGD (full name)-conjugated nanogels carrying docetaxel or paclitaxel were shown to suppress orthotropic breast and pancreatic cancer growth, and their therapeutic efficacy was much superior to that of Abraxane, a clinically-available nanomedicine.

Nanogels are also able to alter the stability in an environment (temperature, pH, and concentration of glutathione)-sensitive manner [20]. Zhu et al. developed a thermally-responsive nanogel based on chitosan and poly (*N*-isopropylacrylamide) blended with acrylamide [21]. This construct was mixed with acrylamide at 5.5% *w*/*w* to increase the volume phase transition temperature from 32 °C to 38 °C and decrease the critical aggregation concentration from 5 µg/mL to 1.1 µg/mL. This nanogel permitted thermally-responsive cellular uptake of the payloads via electrostatic absorptive endocytosis. Importantly, this thermally-responsive nanogel carrying paclitaxel exhibited outstanding anticancer efficacy in HT-29 human colon cancer-bearing xenograft mice. Oh et al. prepared pH-responsive nanogels composed of glycol chitosan grafted with functional 3-diethylaminopropyl groups, which congealed at physiological pH (pH 7.4) and destabilized at the acidic extracellular tumor environment (pH 6.8) due to the protonation of 3-diethylaminopropyl groups [22]. In vitro, this nanogel system demonstrated pH-sensitive release of its payload, doxorubicin, demonstrating that doxorubicin release was significantly accelerated when placed in the acidic medium (pH 6.8) [22]. Chen et al. prepared dual thermo- and pH-sensitive micellar nanogels composed of mPEG-isopropylideneglycerol [23]. First, pH-responsive micelles carrying paclitaxel were formed and then incorporated into the thermosensitive nanogel matrix. This drug delivery system was structured to primarily develop injectable nanogels which stay in a sol-state at room temperature, but forms a gel at body temperature. Paclitaxel incorporated in the micelles with a particle size of 100–200 nm was released at an elevated rate in an acidic medium which mimics the extracellular environment of tumor [23]. Chen et al. used the glutathione gradient (four times greater in concentration in tumors) to reduce disulfide bonds and activate the destabilization of the nanogel [24]. First, sodium alginate was conjugated to doxorubicin via a disulfide linker, and then electrostatically assembled into a nanogel by mixing with aminated superparamagnetic iron oxide nanoparticles. The average particle size of the nanogels was ca. 122 nm with a polydispersity index 0.178. In vitro release profile showed a significantly greater elution profile of the payloads in the medium at higher glutathione concentration, which promises targeted delivery of the payloads, enhanced anticancer efficacy, and low systemic adverse effects in vivo.

## 4. Challenges in Nanogel Development

Although nanogels have been extensively explored as a drug delivery system in the field of academic research in recent years, industrial development of nanogels have not quickly followed. Soni et al. listed a few factors possibly causing the delay of industrial development of nanogels in one of the review articles [25]. These factors include an inefficient transition of in vitro properties to in vivo efficacy, toxicity concerns associated with the degraded gel residue, immunogenicity, discrepancy in pharmacokinetics between rodent models and human, and regulation issues. Hoare et al. also discussed several challenges that remain to improve the clinical applicability of nanogels as a drug delivery system [4]. First, one of the major challenges is the difficulty in handling nanogels in the clinical setting. To ease clinical usage of nanogels, physical gelation should ideally occur at low polymer concentration and at a more precisely-controlled gelation temperature, which reduces risks of premature gelation. Another major challenge is to control the release kinetics of payloads. Development of release rate-modulating nanogels using stimuli-responsive nanogel systems or combinations of polymers with different degradation profiles can be the key solutions to obtain the intended or complex release profiles of payloads from the nanogel matrix. One last major challenge is the practical difficulty in mass production, controllability, or precise preparation of nanogels carrying payloads. One of new strategies that ensures controllable and large-scale production of nanogels is to use a microfluidic technique. Microfluidic technology offers highly-controllable production yields and physicochemical properties in comparison to conventional hydrogel preparation methods. Currently, a number of companies commercializing microfluidic systems (for example, Dolomite) have shown their successful stories in formations of double emulsions, liposomes, polymeric particles, and hydrogels. Especially for hydrogel production, it is well known that microfluidic systems offer excellent controls in size, morphology and, importantly, straightforward scale-up capabilities to produce hydrogels at an industrial scale. Subsequently, there are a number of reports showcasing unique modifications of a microfluidic system to prepare nanogels in a highly-controllable manner [26,27,28]. For example, Agnello et al. employed hyaluronate XS-ethylenediamine-C_18_ dispersions for the production of nanogels using a micromixer microfluidic chip [27]. The nanogels prepared were further lyophilized. The particle size of nanogels (ranged 150–400 nm) increased according to the increase in flow rate. Throughout the process, the fluidity of the polymer dispersions was controlled by using derivatives with different molecular weights or by adding hydroxypropyl-β-cyclodextrins to create molecular complexes with C_18_ chains.

Although some of the challenges involving pharmacokinetics and pharmacological properties still remain unsolved, difficulties in controllable and large amount productions have been overcome or improved using microfluidic systems. When it comes to improving precision and customizability and obtaining high production efficiency, low cost, and convenient operation, 3D printing has recently earned an increasing attention as one of the promising technologies in preparing nanogels for personalized medications.

## 5. Invention of 3D Printing Technology

3D printing is a process of making an object from a digital file. This can be achieved in two ways, subtractive manufacturing and additive manufacturing. In subtractive manufacturing, an object is carved from a big block of materials through the standard machining processes such as drilling, cutting, and milling. In additive manufacturing, successive layers of materials are deposited until the final product is formed. Due to the excess material wastage and limitations in intricate design fabrication, subtractive manufacturing is less widely used. Due to the ability to build complex and intricate designs rapidly, additive manufacturing is vastly used and is now known as a synonym for 3D printing. For 3D printing, a wide variety of materials ranging from hard metals to elastic polymers can be utilized. With the use of different types of materials and rapid prototyping abilities, applications of 3D printing appear to be endless. The areas such as prototyping automotive parts in aeronautical industry, bioprinting implants/organs in medical/health care industry, formulating novel drug delivery systems in pharmaceutical industry, fabricating clothes in textile/fashion industry, food, and chemical industries are representative examples where 3D printing has been widely used.

In 1984, Charles Hull first invented the stereolithography (SLA) technique and patented this 3D printing technology [29]. Hull later co-founded 3D Systems Corporation, which is currently the largest organization producing advanced 3D printers. In 1987, Card Deckard patented the selective laser sintering (SLS) process. Later in 1989, Scott Crump invented and patented fused deposition modeling (FDM) [25]. Crump also co-founded Stratasys Inc., which is another leading 3D printer manufacturing company today. In 1996, three major 3D printers ‘Genisys’ from Stratasys, ‘Actua 2100’ from 3D systems, and ‘Z402’ from Z Corporation were first introduced into the market. These systems were expensive and needed significant improvement. With continuous research and development efforts, decades later, these machines were made inexpensive and are now able to print a wide variety of materials at a higher resolution. With the shift in work flow from mass production to personalization in medical and pharmaceutical industries, 3D printing has gained a great attention in these fields due to its high customizability. In 2006, solid free-form fabrication (SFF) technique, a 3D printing technique similar to the inkjet printing, was used by Wang et al., to fabricate zero-order release pharmaceutical dosage forms [30]. In March 2014, Aprecia Pharmaceuticals have filed a patent for 3D printed rapid disintegrating dosage form [31]. Later in July 2015, FDA approved the first 3D printed tablets called Spritam^®^ (levetiracetam) produced by Aprecia Pharmaceuticals [32].

## 6. Types of 3D Printing

Depending on the process and type of materials used, 3D printing can be broadly classified into stereolithography (SLA), selective laser sintering (SLS), fused deposition modeling (FDM), inkjet, and laminated object manufacturing (LOM). SLA is the oldest 3D printing technique and the first to be commercialized. SLA machines work on a principle called photopolymerization, where a liquid polymer is cured into a solid or a semi-solid form at precise locations using a laser beam [33]. This process is continued in layer-by-layer fashion until the final product is formed. These machines have a platform which can move in the z-axis. A thin layer of photopolymer is laid from the reservoir on to this platform and laser beam cures the polymers in designed shape. The building platform moves down along the z-axis and the steps are repeated until the object is formed. Objects made using SLA are very precise and high in resolution. However, the finished objects need to be cleaned and a secondary curing of the finished object by laser beam is often necessary. Currently available photopolymers are expensive and, over time, they solidify and become brittle. Additionally, this technology can be utilized only with photopolymers and is limited by the variety of photo-resins.

SLS is similar to SLA, except that the materials used in this process are in a powder form. A high-power laser beam sinters the powder in selected pattern and fuses it to the layers below. The powder bed drops down along the z-axis and rollers lay down a thin layer of powder onto the platform. The laser again fuses the powder to the layers below and this continues until the final product is formed. The powder bed itself acts as a supporting material for the object and, hence, this form of 3D printing is well known for building complex geometries and structures. A wide range of metals, ceramics and plastics in a powder form can be used for 3D printing using this technique. The objects made from SLS have higher mechanical strength than any other 3D printing technologies. Due to this reason, SLS is widely used in industries to prototype machine parts. However, the powder sintering takes a high amount of energy and high temperatures making the 3D printer expensive, and it requires extra time for cooling and cleaning the finished product.

FDM-type 3D printers are also known as extrusion-based or fused-filament fabrication/free-form fabrication (FFF)-based 3D printers. FDM printers melt down a thermoplastic filament onto the building platform in a layer-by-layer pattern until the object is built. These printers have printer-heads which melt the thermoplastic filaments and move along the x- and y-axes laying the molten polymer in the shape of a required object on the building platform. These printers are comparatively inexpensive and widely used nowadays. FDM printers are also popular due to their ability to print small objects with complex geometries at a high resolution. The materials used for FDM printers are usually industrial-grade, and the printed objects are durable, showing excellent chemical, thermal, and mechanical properties. ABS (acrylonitrile butadiene styrene), PLA, and nylon are the most commonly used thermoplastics for FDM printing.

Inkjet 3D printers are also known as drop-on-demand (DOD), binder jetting, or material jetting 3D printers. In the process of printing, a thin layer of powder is laid on the surface of the building platform and the print-head selectively deposits the binding liquid onto the powdered bed, binding the particles together. A second layer of powder is laid on to the platform, and the process is repeated. This iteration of the process is done in a layer-by-layer fashion until the required object is built. This technique allows to fabricate multi-color objects and also to prototype very small objects with a much higher accuracy. However, the resulting constructs lack smooth surfaces and are often brittle, requiring post-processing.

The LOM-type of 3D printing was first developed by Helisys Inc. In the printing process, adhesive coating materials (papers, plastic films, or thin laminates of metals) are fused together using external pressure or heat. A laser beam cuts these layers into the shaped objects. Printed objects often requires post-process drilling, sanding, and machining. LOM-type 3D printers are inexpensive, fast to print objects, and the materials used for printing are also inexpensive. However, these machines are very large in size and produce excess material waste. These machines are widely used by arts and crafts product developers.

For 3D printing an object, 3D modeling is the first step. Computer aided design (CAD) software is used to make a 3D object. AutoCAD, Maya, and SolidWorks are some of the commercially available CAD software. Blender, SketchUp, and FreeCAD are some of the free CAD software for the beginners. 3D scanning an object can also produce 3D models. For a 3D printer to read the files, 3D models are converted into the STL (standard tessellation language) file format by these CAD software packages. This file format was first developed and used by 3D Systems for their SLA machines. Currently, most of the available 3D printers uses this file format system to read and print a 3D object. These STL files are then sliced into thin horizontal slices by slicing software. Slic3R, ReplicatorG, and Makerware are some of the commonly-used slicing software programs. These software packages assign the tool path for the print-head. It dictates to the 3D printer the speed, height of the layers, temperature, type of the material used, and particular geometry if supporting material is needed for printing. 3D printers receive the instructions from the slicing software and start building the object in a layer-by-layer fashion.

## 7. 3D Printing in Pharmaceutical Sciences

**Figure 2:**
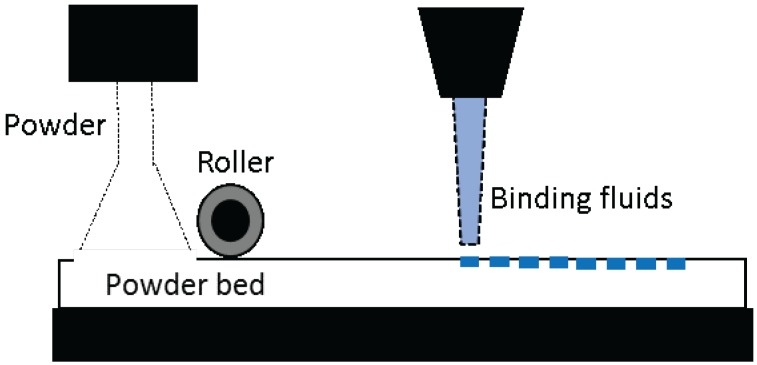
Zipdose^®^ Technology developed by Aprecia Pharmaceuticals.

Using 3D printing technology, various oral dosage forms can be fabricated. Spritam^®^, a rapidly-disintegrating oral dosage form of leveltiracetam, was the first 3D-printed dosage form approved by the FDA in August 2015 [34]. This dosage form uses Zipdose^®^ technology (Aprecia Pharmaceuticals, Langhorne, PA, USA), which is similar to inkjet-based 3D printing (Figure 2).

Its approval by the FDA has stimulated interests in 3D printing in pharmacy and promises advances in this field. SFF, also known as 3D printing, was first used by Wu et al. to fabricate controlled drug delivery systems [35]. Their team fabricated 3D structures using poly (caprolactone) (PCL) and PEO as biomaterials. Methylene blue and alizarin yellow were used as sample drug molecules to demonstrate the ability of fabricating complex drug delivery systems using 3D printing technology. The 3D printing technology used by these researchers is similar to inkjet printing that uses mixture of PCL and PEO as a powder base and chloroform as a binder solution. To ensure complete removal of chloroform, fabricated dosage forms were vacuum-dried for seven days. Release of two dyes showed two-pulse release patterns attributed to dye distribution in the polymer matrix incorporated in a matrix device [35]. Rowe et al. also demonstrated the ability to fabricate various complex oral dosage forms using 3D printing technology. Oral dosage forms with various drug release patterns including immediate-extended release tablets (stimulated via different pH-based release mechanism), breakaway tablets, enteric dual pulsatory tablets, and dual pulsatory tablets (utilizing opposite pH-based solubility of two excipient sections) were fabricated using inkjet-type 3D printing technology [36]. Using different copolymers of Eudragit^®^ as excipients, the different tablets mentioned above, which contain diclofenac as a sample drug, were conveniently fabricated using 3D printing technology, which demonstrated various (desired) drug release patterns in vitro. Results from these findings support the feasibility of 3D printing in manufacturing smart oral dosage forms with various drug release patterns [36]. Using excipients, such as colloidal silicone dioxide, polyvinylpyrrolidone, lactose, and mannitol, Yu et al. were able to fabricate fast-dissolving oral tablets containing acetaminophen using inkjet-based 3D printing technology [37]. Here, faster drug release from 3D printed tablets were achieved compared to conventionally-compressed tablets. 3D printed tablets released 98.5% of acetaminophen in 2 min, while compressed tablets took ca. 20 min to release a similar amount of drug. Drug release and pharmaco-technical properties of 3D printed tablets were acceptable, but the tablets had poor friability [37]. Tablets with zero-order release kinetics were 3D printed (using inkjet printing method) by Yu et al. using acetaminophen as an active drug and a few excipients including hydroxypropyl methyl cellulose, ethyl cellulose, Eudragit^®^, and stearic acid [38]. Here, tablets were printed in three dimensions in a gradient pattern to limit the release of the drug only from the side walls of the tablet [38]. This study clearly demonstrated the ability of 3D printing to fabricate objects with complex structures and functional properties. A complex multidrug dosage form with immediate, controlled, and sustained release compartments carrying aspirin, hydrochlorothiazide, pravastatin, atenolol, and ramipril was 3D-printed [39]. This study took personalized medicine into a new direction, especially for patients who need to take a number of drugs to treat multiple medical conditions (especially elderly patients suffering from multiple diseases). Instead of taking multiple pills, one tablet can be taken for minimizing patient errors [39]. There are several other studies conducted to prepare core-shell structured tablets or intragastric floating tablets: Using polyvinyl alcohol as an excipient, a unique oral dosage form was designed carrying paracetamol and caffeine. This tablet had alternating layers and a core-shell (DuoCaplet) structure carrying two drugs [40]. Chai et al. were able to 3D-print tablets (FDM technology) for intragastric floating delivery of domperidone using hydroxypropyl cellulose as an excipient.

## 8. Fabrication of Hydrogels and Nanogels Using 3D Printing Technology for Pharmaceutical and Biomedical Applications

Increasing demands for developing personalized medicines and specialized drug delivery systems have motivated researchers in the biomedical field to fully utilize 3D printing technology. 3D printing has not yet been heightened by its capabilities in the biomedical field, however, there are great benefits that 3D printing technology can bring up to the table when it comes to the precise and convenient production of small and complex objects.

When a tissue or organ fails, transplantation from a living or dead donor has been the only treatment option. However, there has been a constant shortage of tissues/organs available from donations. Moreover, finding a right match to the host is often a difficult task and is a pinnacle reason for surgery failure. Recent advances in biomaterials that can be 3D-printed have revolutionized the traditional tissue engineering and regenerative medicine approach, which can avoid organ shortage problems. Hydrogels are known to provide excellent environment for cells and so are commonly used as cell carriers for 3D bioprinting [41]. Alginic acid [42], chitosan [43], hyaluronic acid [44], agarose [45], and PEG [46] are some of the hydrogel materials used for bioprinting. Using 3D printing technology, hydrogels loaded with cells are laid in a layer-by-layer fashion in the shape of an organ and placed in a bioreactor until fully grown [41,47]. To fabricate a complex organ, multiple print heads loaded with different types of cells/biomedical carrier materials can be used [48]. Although there is a great advance in bioprinting, vascularization has been the greatest challenge for 3D bioprinting thick and complex organs [49,50]. One of the promising approaches is precise placement of vascular cells while bioprinting a complex organ [51]. Uses of sacrificial materials (dissolvable polymers) [52] and spatial micropatterning of photo-lithographized polymers [53], are some of the other vascularization strategies currently used in 3D bioprinting. Researchers have used bioprinting technology to fabricate heart valves [47], urethra [54], ovaries [55], bionic ears [56], and bone and cartilage [41]. Although many researchers have already provided a proof-of-concept to fabricate living organs from a host’s cells, bioprinting a fully-functional human organ is still in its early stages of development.

**Figure 3:**
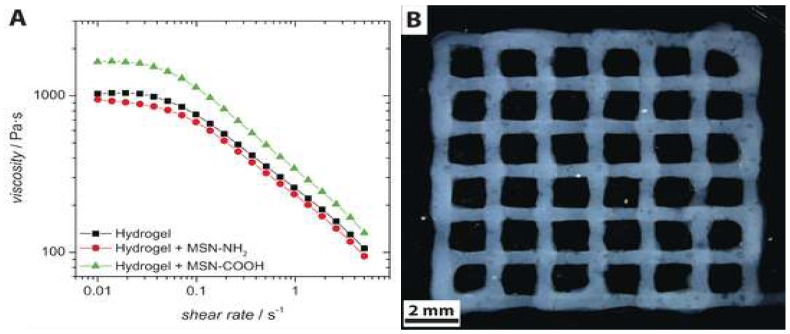
(**A**) Viscosity over shear rate for different systems. (**B**) Stereomicroscopic image showing a top view of a four-layer construct (obtained from [57]).

3D printing has been naturally recognized as a practical technique to create 3D structured nanogels. Most of the nanogels were 3D-printed in a structure of drug(s)/photoinitiator-loaded nanoparticles, liposomes, or nanoemulsions suspended in hydrogels. In this way, hydrophobic drugs can be incorporated in the nanocarriers and their elution into the medium can be extended for longer periods of time due to the drug molecules passing through two carriers, nanocarriers and hydrogel matrix. For example, Baumann et al. first formed spherical mesoporous silica nanoparticles functionalized with either amino or carboxyl groups resulting in formations of positively- or negatively-charged particles which serve as drug carriers (Figure 3). Both nanoparticles were then incorporated in 3D-printed hyaluronic acid-based hydrogel scaffolds [57]. Nanoparticles incorporated in the hydrogel matrix did not significantly alter rheological properties of 3D-printed hydrogels, but electrostatic interactions between nanoparticles and hyaluronic acid (negatively charged) determined the drug release kinetics of nanoparticles, which was faster for the nanoparticles functionalized with carboxyl groups than those functionalized with amino groups.

**Figure 4:**
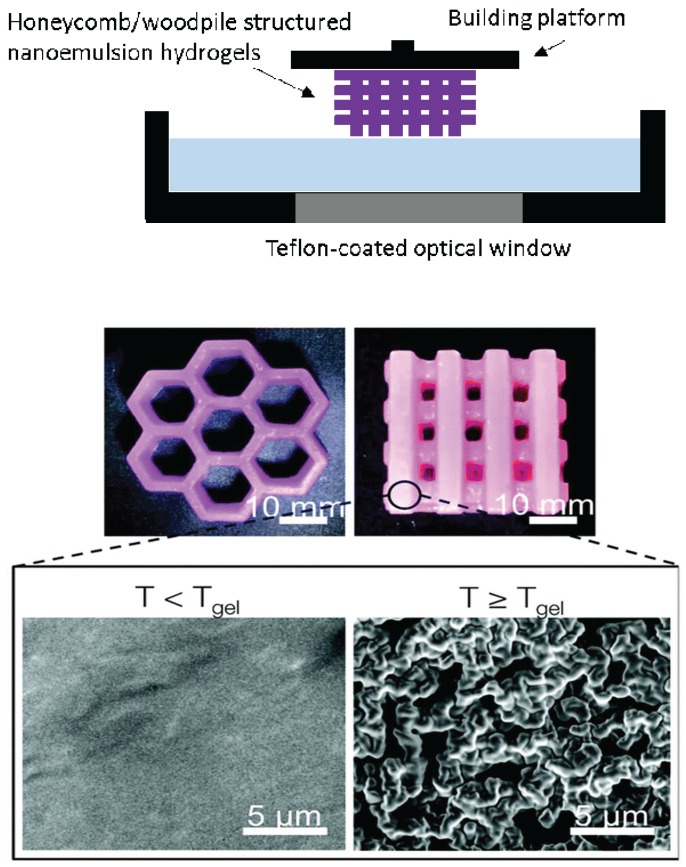
SLA 3D printing of honeycomb and woodpile structured hydrogels using thermoresponsive nanoemulsion inks (modified from [59]).

Nanogels carrying a photoinitiator were 3D-printed to improve gelation process or mechanical strength of the printed hydrogels and provide local delivery of therapeutic entities. Pawar et al. 3D-printed hydrogels carrying photoinitiator-containing nanoparticles [58]. The photoinitiator plays an important role as one of the major determinants for the quality of the printed object and time required for fabrications. Among various types of photoinitiators, 2,4,6-trimethylbenzoyldiphenylphosphine oxide (TPO) has been known to be the most efficient in initiating the free radical polymerization of various monomers with a high molar extinction coefficient; however, very low water solubility of TPO (3.13 µg/mL) has been a problem preventing it from being widely used. In this study, TPO was first encapsulated in nanoparticles (particle size of 180–350 nm; polydispersity index of 0.02–0.2) formed by spray drying of volatile microemulsions, and enabled rapid 3D printing of complex hydrogel structure in water (without addition of organic solvent or chemical modifications) using SLA-based low-cost 3D printers. 3D printing of hydrogels was performed at a rate of 6 s per layer (100 µm layer thickness). A woodpile-structured hydrogel object containing 80% (*w*/*w*) water content was built within 25 min. The mechanical strength of the printed hydrogels was ca. six times greater than one of the commercially available reference hydrogels, poly (ethylene glycol) diacrylate hydrogels, which is a great property for implant purposes. Hsiao et al. 3D-printed hierarchical mesostructured hydrogels carrying nanoemulsions (Figure 4) [59]. Nanoemulsions consist of a continuous phase with poly (ethylene glycol) dimethacrylate and sodium dodecyl sulfate dissolved in water and an oil phase consisting of poly (dimethyl siloxane) droplets. Nanoemulsions were then mixed with a photoinitiator, TPO, placed in the resin tank and heated to induce self-assembly throughout interdroplet bridging of poly (ethylene glycol) dimethacrylate. A commercially-available SLA printer with a custom-modified Teflon window for enhancing oxygen permeation was used to 3D print honeycomb- and woodpile-structured hydrogels. The 3D-printed object allows for the transport of molecules and mesoscopic objects. They emphasized that nanoemulsions can serve as self-assembling precursor inks to be rapidly photopolymerized into macroscale shapes with the desired internal features.

Nanogels have been also 3D-printed for bioprinting in tissue engineering [60]. Pluronic F-127 gels resemble the native tissue but do not last long enough for long-term cell culture. Muller et al. mixed unmodified Pluronic F-127 with acrylated Pluronic F-127 to create strong and stable nanostructured hydrogels via UV crosslinking [61]. By subsequent elution of the unmodified Pluronic from the crosslinked networks, cell viability of the encapsulated chondrocyate was increased. The mechanical strength of the nanostructured hydrogels was further increased by adding methacrylated hyaluronic acid.

**Figure 5:**
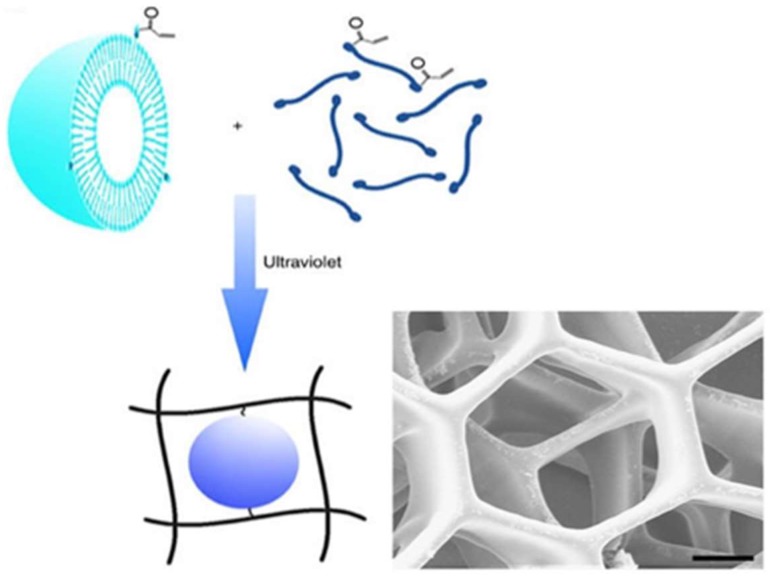
Schematic presentation of installing PDA nanoparticles in the network of hydrogels and scanning electron microscope image of the nanogel detoxifier (modified from [62]).

3D-printed nanogels were utilized as a detoxification device [62]. Gou et al. 3D-printed a photo-crosslinked poly (ethylene glycol) diacrylate hydrogel as a 3D matrix and a polydiacetylene (PDA) nanoparticles (the mean particle size of 100 nm with polydispersity index of 0.23) possessing acrylamide groups were chemically tethered via polymerization and installed in the hydrogel matrix (Figure 5). The authors were able to 3D-print hydrogels in the shape of the liver lobules. Due to the extremely slow degradation rate of the hydrogels as the nanoparticles were chemically immobilized in the hydrogel matrix, unintended premature release of nanoparticles was not observed. PDA nanoparticles were able to attract, capture, and sense toxins while the hydrogel matrix allowed toxins to be trapped, which can serve as a bio-inspired detoxification device for liver-failure patients.

Nanogels have been used as a filler of the 3D-printed external construct to modify its physicochemical properties or biocompatibilities. Liu et al. incorporated Poloxaner 407 (also known as Pluronic F-127) nanogels carrying simvastatin into the 3D-printed porous titanium alloys for orthopedic applications [63]. Titanium alloy by itself was poorly compatible with bone ingrowth. Poloxamer 407 nanogels carrying simvastatin induced osteogenic factors and promoted osteogenesis, but are poor in mechanical strength [63]. By combining two composites, 3D-printed porous titanium scaffolds filled with nanogels carrying simvastatin achieved both good compatibility with bone ingrowth and mechanical strength of the construct. As a result, this construct significantly enhanced vascularization and presented a correlation between the volume of new bone and neovascularization in 4–8 weeks after implantation.

3D printing can be used to create a mold or a frame for fabricating nanogels. Tao et al. 3D-printed a mold for preparing nanogels using the MRI of a patient’s brain tumor cavity [64]. This “customizability” is one of the unique benefits that 3D printing technology can offer to the indirect production of nanogels in customer-based settings. In this study, customized conformal hydrogel nanocomposites containing paclitaxel were prepared which has a shape of the patient’s glioma tumor cavity created after surgery [64]. For nanogels, paclitaxel was encapsulated in nanoparticles and then suspended uniformly in macroporous hydrogels. It was observed that nanoparticles slowly released paclitaxel, and hydrogel matrix further delayed elution of paclitaxel into the external medium in vitro. In cell culture settings, slower release of paclitaxel efficiently inhibited the proliferation of glioma tumor cells, promising an efficient use of hydrogels as a cavity filler after surgical tumor resection to eradicate any residual tumor tissues/cells.

The following example employed “micro” gels (which is larger in particle size than nanogels) but is worth mentioning due to the unique approach of utilizing microgels for 3D printing objects: O’Bryan et al. 3D-printed silicone elastomers into the micro-organogel support medium (light mineral oil, polystyrene-*block*-ethylene propylene diblock copolymer and polystyrene-*block*-ethylene/butylene-*block*-polystyrene triblock copolymer, forming microgels composed of packed micelles) [65]. This micro-organogels allowed more precise 3D-printing permitting fabrication of thin-walled tubes with a thickness of 450 µm that were robust enough to be handled and removed from the supporting material.

## 9. Conclusions

3D printing of nanogels are yet to be explored more in the fields of academic and industrial research. Microfluidic fields have been successfully utilized for large-scale productions of nanogels. 3D printing can offer additional benefits to the productions of nanogels, including high precision, high production efficiency, low cost, convenient operation, and customizability. 3D-printed nanogels that may carry various therapeutic entities and biomedical materials are anticipated to be widely used in wound healing, regenerations of tissues/organs, detoxification, medical implants, local disease treatments, and personalized medications in the near future. 3D printing of nanogels will also permit rapid prototyping of soft anatomical models for preoperative surgical planning in cardiovascular, neuro, and visceral surgeries.
